# Diagnostic accuracy of the WHO clinical staging system for defining eligibility for ART in sub-Saharan Africa: a systematic review and meta-analysis

**DOI:** 10.7448/IAS.17.1.18932

**Published:** 2014-06-12

**Authors:** Chigomezgo Munthali, Miriam Taegtmeyer, Paul G Garner, David G Lalloo, S Bertel Squire, Elizabeth L Corbett, Nathan Ford, Peter MacPherson

**Affiliations:** 1 Department of International Public Health, Liverpool School of Tropical Medicine, Liverpool, UK; 2 Department of Clinical Sciences, Liverpool School of Tropical Medicine, Liverpool, UK; 3 TB and HIV Group, Malawi–Liverpool–Wellcome Trust Clinical Research Programme, Blantyre, Malawi; 4 Department of Clinical Research, London School of Hygiene and Tropical Medicine, London, UK; 5 Department of HIV/AIDS, World Health Organization, Geneva, Switzerland

**Keywords:** HIV, WHO clinical staging system, antiretroviral therapy, sub-Saharan Africa, systematic reviews, CD4 cell count

## Abstract

**Introduction:**

The World Health Organization (WHO) recommends that HIV-positive adults with CD4 count ≤500 cells/mm^3^ initiate antiretroviral therapy (ART). In many countries of sub-Saharan Africa, CD4 count is not widely available or consistently used and instead the WHO clinical staging system is used to determine ART eligibility. However, concerns have been raised regarding its discriminatory ability to identify patients eligible to start ART. We therefore reviewed the accuracy of WHO stage 3 or 4 assessment in identifying ART eligibility according to CD4 count thresholds for ART initiation.

**Methods:**

We systematically searched PubMed and Global Health databases and conference abstracts using a comprehensive strategy for studies that compared the results of WHO clinical staging with CD4 count thresholds. Studies performed in sub-Saharan Africa and published in English between 1998 and 2013 were eligible for inclusion according to our predefined study protocol. Two authors independently extracted data and assessed methodological quality and risk of bias using the Quality Assessment Tool for Diagnostic Accuracy Studies (QUADAS-2) tool. Summary estimates of sensitivity and specificity were derived for each CD4 count threshold and hierarchical summary receiver operator characteristic curves were plotted.

**Results:**

Fifteen studies met the inclusion criteria, including 25,032 participants from 14 countries. Most studies assessed individuals attending ART clinics prior to treatment initiation. WHO clinical stage 3 or 4 disease had a sensitivity of 60% (95% CI: 45–73%, *Q*=914.26, *p*<0.001) and specificity of 73% (95% CI: 60–83%, *Q*=1439.43, *p*<0.001) for a CD4 threshold of ≤200 cells/mm^3^ (11 studies); sensitivity and specificity for a threshold of CD4 count ≤350 cells/mm^3^ were 45% (95% CI: 26–66%, *Q*=1607.31, *p*<0.001) and 85% (95% CI: 69–93%, *Q*=896.70, *p*<0.001), respectively (six studies). For the threshold of CD4 count ≤500 cells/mm^3^ sensitivity was 14% (95% CI: 13–15%) and specificity was 95% (95% CI: 94–96%) (one study).

**Conclusions:**

When used for individual treatment decisions, WHO clinical staging misses a high proportion of individuals who are ART eligible by CD4 count, with sensitivity falling as CD4 count criteria rises. Access to accurate, accessible, robust and affordable CD4 count testing methods will be a pressing need for as long as ART initiation decisions are based on criteria other than seropositivity.

## Introduction

At the end of 2012, an estimated 9.7 million HIV-positive patients had initiated antiretroviral therapy (ART) worldwide, a 30-fold increase since 2003 [1]. Despite this rapid scale-up, 39% (under 2010 World Health Organization guidelines) and 66% (under 2013 WHO guidelines) of all ART-eligible individuals are still not taking ART in low- and middle-income countries [1]. Mathematical modelling [2] and observational studies [3] suggest that increasing population ART coverage could dramatically reduce HIV transmission and mortality. The landmark HTPN052 trial showed a 96% reduction in HIV transmission to uninfected partners in serodiscordant couples with immediate ART initiation [4]. On the basis of this evidence, and data supporting clinical benefit, World Health Organization (WHO) has recently issued new ART guidance which recommends starting ART in HIV-positive adults with a CD4 count ≤500 cells/mm^3^ [1].

Measurement of CD4 count is the preferred method for ART eligibility assessment in HIV-positive patients. However, in sub-Saharan Africa, this is still not widely available [5]. For example, in 2012 only 11% of ART centres in Malawi had a functioning CD4 count machine [6]. Even where CD4 count measurement is available, quality may not be assured and access to this service can be unreliable, requiring multiple expensive visits to healthcare facilities [7]. This contributes to the high rates of pre-ART loss to follow up seen in many ART programmes [8–10]. Point of care CD4 count testing has the potential to overcome some of these barriers [11] but has not been widely rolled out in Africa.

Where CD4 count is not immediately available, the WHO clinical staging system is used for initial assessment of ART eligibility. This clinical staging system was developed in 1990 based on original clinical case definitions for AIDS [12] and was intended as a clinical method for diagnosing HIV infection and monitoring disease progression [13, 14]. It has four clinical stages, numbered stage 1 to stage 4. The clinical stages are hierarchical with standardized clinical parameters; stage 1 is primary HIV infection, while individuals in stage 4 have advanced HIV disease or AIDS. Stage-defining conditions are used to classify patients into one of the four clinical stages [15].

Over the years, the WHO clinical staging has undergone modifications. It was first revised in 2005 [16]. The current version, revised in 2009, has 40 clinical conditions [17]. Under the 2013 WHO ART guidelines, adults in WHO stage 3 or 4 are considered eligible to initiate ART [1]. As well as being used to identify individuals in need of ART, although not yet validated, WHO staging can be used in setting with limited CD4 count availability to provide a measure of programme performance by identifying patients who have presented late for treatment [6].

Although the WHO clinical staging system has been widely adopted as a tool for assessing ART eligibility, healthcare workers find it time-consuming, complex and poorly applicable in resource-poor settings without access to sophisticated diagnostics [18]. Moreover, its performance as an ART eligibility assessment tool has not been evaluated. We undertook this systematic review in order to evaluate the accuracy of the WHO clinical staging system in identifying ART eligibility as defined by CD4 count thresholds.

## Methods

### Inclusion and exclusion criteria

The review included studies published between 1 January 1998 (when combination ART became available in resource-limited settings) and 25 May 2013. We sought studies in which WHO clinical stage and CD4 count were contemporaneously measured in the same HIV-positive patient during ART eligibility assessment. Studies were eligible for inclusion if they presented data on the WHO clinical staging system as the index test and CD4 count as the reference test at CD4 count cut-offs of either ≤200, ≤350 or ≤500 cells/mm^3^, the three main cut-offs used to define ART eligibility in international guidelines over the past decade. Randomized controlled trials, cohort studies, cross sectional studies and studies that analyzed routinely collected data were included. For studies where the primary objective was not to evaluate the performance of the WHO clinical staging system, but where a subgroup of participants met inclusion criteria, only data from this subgroup were extracted for analysis. Studies conducted in adults (≥15 years old) in sub-Saharan Africa were included; children who were excluded as different criteria are used for assessing treatment eligibility. Studies were limited to sub-Saharan Africa to improve comparability. Studies that enrolled only participants in a particular WHO clinical stage were also excluded as they presented insufficient data for calculating test performance.

### Search strategy

We searched PubMed and Global Health [19] databases using a comprehensive search strategy following a protocol (CRD42013004801) registered under the University of York database for Prospectively Registered Systematic Reviews in Health and Social Care (PROSPERO) [20] (Supplementary file). Search terms and full search strategies for both databases are shown in Appendices 1 and 2. We used MeSH terms, free text and keyword terms: HIV infection; Acquired Immunodeficiency Syndrome; CD4 Lymphocyte Count; WHO; WHO clinical staging; Africa south of the Sahara; poverty; developing countries. The search was limited to articles published in English. In addition to database searches, efforts were made to identify any additional articles that could have been missed by screening the bibliographies of papers initially identified as eligible for the study. We also systematically searched through conference abstracts from the International AIDS Society conference (IAS) and the Conference on Retroviruses and Opportunistic Infections (CROI) from 1998 to 2012.

The Preferred Reporting Items for Systematic Reviews and Meta-Analyses (PRISMA) guidelines were followed [21] (Supplementary file). After exclusion of duplicate studies, two reviewers (CM and PM) independently screened the titles and abstracts of all retrieved studies to identify studies eligible for full text review. Discrepancies were resolved by group discussion with a third reviewer (MT). Two reviewers (CM and PM) then independently reviewed the full text of selected studies. A consensus meeting was held with a third reviewer (MT) to determine final study inclusions.

Two reviewers (CM, PM) independently extracted data from included studies. For each study, the following study information was extracted: study setting (hospital, outpatient clinic, antenatal care or community); type of participants (ART-naïve or reinitiating treatment); cadre of health worker performing WHO clinical staging; whether health workers had formal study pre-training on how to perform WHO clinical staging assessment; availability of support and routine supervision from HIV clinical experts during the study period; and whether the health worker performing WHO clinical staging referred to a reference checklist of the WHO clinical staging system while performing staging. Corresponding authors of included studies were contacted by email to provide data where necessary.

To assess the accuracy of the WHO clinical staging system at the three predefined CD4 count thresholds, the same two reviewers independently extracted data on the number of ART eligible participants who were classified as: true positive (those correctly identified as eligible for ART by WHO staging), false positive (those that were classified as being eligible for ART by WHO staging but did not require ART following CD4 count testing), true negative (those correctly identified as not eligible for ART by WHO staging) and false negative (those that were wrongly classified as not eligible for ART by WHO staging). Data were then summarized in two-by-two tables. Sensitivity, specificity, positive predictive values and negative predictive values were calculated using CD4 count thresholds as the referents and 95% confidence intervals were calculated for each statistic. The WHO clinical staging system has undergone minor incremental changes since its inception. However, no study specified which version of the staging system was used, so analysis was not stratified by the WHO staging system version.

### Statistical analysis

For each CD4 count threshold, pooled sensitivity and specificity were calculated using bivariate hierarchical random effects models [22]. Heterogeneity was assessed by the Q statistic. Coupled forest plots showing sensitivity and specificity, and hierarchical summary receiver operational curves (HSROC) were generated. All analyses were done using Stata version 12.1 (Statacorp, College Station, TX).

### Quality and risk of bias of selected studies

The Quality Assessment Tool for Diagnostic Accuracy Studies (QUADAS-2) was used to assess the methodological quality of included studies and to inform judgements about risk of bias [23]. We modified the original QUADAS-2 tool by omitting two items as they were not relevant and applicable to the study. The omitted items were: whether participants received the same reference standard regardless of the index test result; and whether the index test was part of the reference test.

Two reviewers (CM and PM) independently applied nine QUADAS-2 signalling questions across four domains to make an overall risk of bias judgement for each study. The domains were: selection; index test conduct; reference test conduct; and participant flow and timing. As per QUADAS-2 guidelines, a study was judged to be at an overall low risk of bias if responses to all signalling questions across four domains gave no concerns about bias. If a study had one or more signalling questions where the response indicated likely bias, it was judged to be at high risk of bias. A judgement of “unclear” was given where inadequate information to assess risk of bias was available.

## Results

### Study characteristics

**Figure 1:**
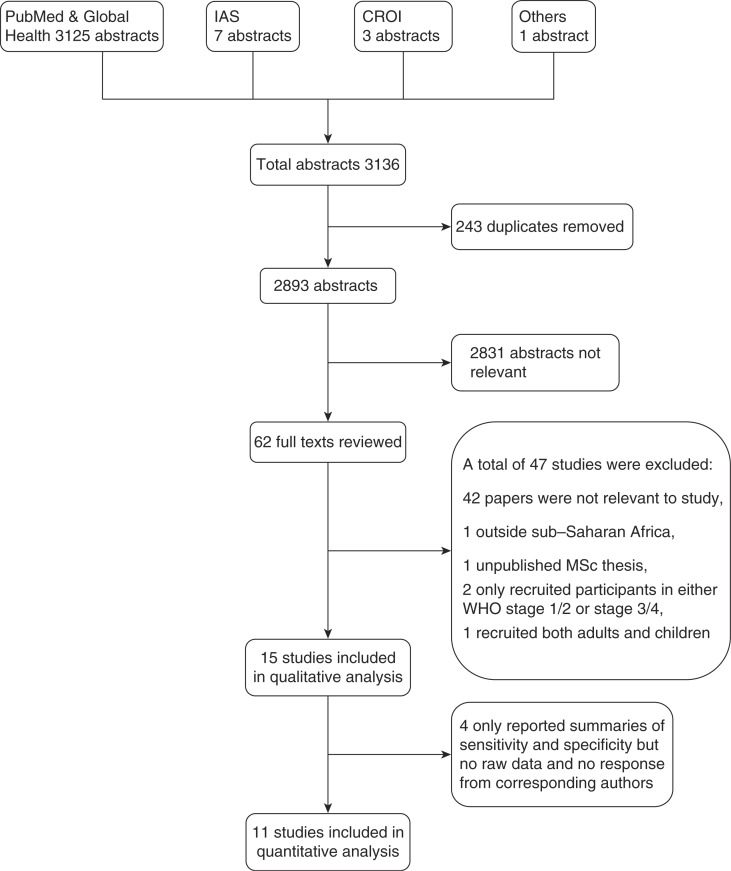
PRISMA flowchart summarizing articles identified through search strategy and conference abstracts. IAS, International AIDS Society conference; CROI, the Conference on Retroviruses and Opportunistic Infections.

The search identified 2893 unique abstracts, of which 15 studies including 25,032 participants met the study inclusion criteria (Figure 1). The characteristic of included studies are summarized in Table 1. Eleven of the 15 studies were from East Africa, two studies were from Southern Africa, one study was from West Africa, and one study was a multicentre study conducted in eight African countries. Studies’ year of publication ranged from 1999 to 2011.

**Table 1:** Characteristics of included studies

Study	Country	Study design	Description of study design	Study period	Population	Number of participants assessed
Baveewo *et al*. [25]	Uganda	Cross sectional	Newly diagnosed HIV-positive patients referred to ART clinic.	January 2007 to April 2007	ART^a^ clinic attendees	395
Boniphace *et al*. [26]	Tanzania	Cross sectional	Newly diagnosed HIV-positive patients referred to ART clinic.	April 2008 to December 2008	ART clinic attendees	421
Carter *et al*. [27]	Cameroon, Cote d'Ivoire, Kenya, Mozambique, Rwanda, South Africa, Uganda, Zambia, Thailand.	Prospective cohort	Participants were antenatal and postnatal women screened for ART eligibility as part of PMTCT programme	January 2003 to March 2008	Antenatal and postnatal women in PMTCT^b^ programme	6036
French *et al*. [34]	Uganda	Prospective cohort	Newly diagnosed HIV-positive patients referred to ART clinic.	October 1994 to January 1995	ART clinic attendees	201
Fox *et al*. [28]	South Africa	Prospective cohort	Small study in a large clinical trial evaluating nurse vs. doctor ART model of care delivery.	January 2009	ART clinic attendees	812
Ilovi *et al*. [33]	Kenya	Cross sectional	Recruited newly diagnosed HIV patients in the outpatient clinics and hospital inpatients.	April 2010 to February 2011	Medical inpatients and HIV clinic attendees	152
Jaffar *et al*. [35]	Uganda	Cross sectional	Newly diagnosed HIV-positive patients referred to ART clinic.	August 2004 to December 2006	ART clinic attendees	4321
Kagaayi *et al*. [36]	Uganda	Prospective cohort	Recruited participants from an on-going cohort study	NR^c^	ART clinic attendees	1221
Kassa *et al*. [24]	Ethiopia	Cross sectional	Participants were enrolled from community and hospital inpatients medical wards.	November 1996 to April 1997; February 1997 to April 1998	ART clinic attendees	167
Martinson *et al*. [37]	South Africa	Cross sectional	Newly diagnosed HIV-positive patients referred or walked-in to ART clinic.	NR	ART clinic attendees	2072
McGrath *et al*. [29]	Malawi	Cross sectional	Newly diagnosed HIV patients in the community	From June 2005	People in the community	150
Miiro *et al*. [30]	Uganda	Prospective cohort	Recruited participants from an on-going cohort study	October 1995 to April 2006	ART clinic attendees	2892
Morpeth *et al*. [31]	Tanzania	Cross sectional	Newly diagnosed HIV-positive patients referred to ART clinic.	August 2004 to June 2005	ART clinic attendees	202
Tassie *et al*. [32]	Malawi	Cross sectional	Newly diagnosed HIV-positive patients referred to ART clinic.	NR	ART clinic attendees	206
Torpey *et al*. [38]	Ghana	Retrospective review of routine collected data	Participants were HIV patients receiving ART in 6 public ART clinics.	200–2005	ART clinic attendees	5784

a ART: antiretroviral therapy.

b PMTCT: prevention of mother to child transmission of HIV.

c NR: not reported.

More women than men were enrolled in all of the studies except one [24]; the proportion of women ranged from 53 to 100% of the study population. Eight studies [25–32] only enrolled participants who were ART-naïve; the other studies did not state whether participants were ART-naïve or reinitiating ART.

### Study setting

Twelve of the 15 studies enrolled participants who were newly diagnosed HIV-positive patients being assessed in ART clinics after HIV testing and counselling (HTC) (Table 1). The three other studies enrolled participants from a rural community as part of a demographic and health survey (DHS) [29], an antenatal clinic as part of a PMTCT programme [27] and patients admitted in medical wards and assessed in an outpatient medical assessment unit [33].

### Quality and risk of bias assessment

There was considerable variation in the methodological quality of included studies (Table 2). Only one study was judged to be at overall low risk of bias [25] and most studies (*n*=11) had an unclear overall risk of bias as there was insufficient description of one or more items. Of particular concern was the high numbers of studies that did not provide sufficient description of conduct of WHO clinical staging assessments including whether training was provided, whether checklists were used and whether individuals who performed staging were blinded to CD4 count results (Table 3).

**Table 2:** Methodological quality and risk of bias assessment of included studies

Domains	Participant selection		Conduct of index test (WHO clinical staging system)		Conduct of reference test (CD4 count measurement)		Participant flow and timing			
					
Signalling questions	Did study enrol sample of participants representative of the wider population?	Were there clear participant selection criteria, avoiding inappropriate exclusions?	Did the study clearly describe how WHO staging was performed?	Were index test results interpreted without knowledge of results of the reference standard?	Did the study describe how CD4 count testing was done?	Is the reference test likely to correctly classify ART eligibility?	Was the time period between staging and CD4 count testing short enough not to affect participant stage?	Did all participants receive the same reference standard?	Were all participants included in the analysis?	Overall risk of bias judgement
Baveewo *et al*. [25]	Yes	Yes	Yes	Yes	Yes	Yes	Yes	Yes	Yes	Low
Boniphace *et al*. [26]	Yes	Yes	Unclear	Unclear	Yes	Yes	Unclear	Yes	Yes	Unclear
Carter *et al*. [27]	Yes	Unclear	Unclear	Unclear	Unclear	Yes	No	Unclear	Yes	High
French *et al*. [34]	Yes	Yes	Unclear	Yes	Yes	Yes	Unclear	Yes	Yes	Unclear
Fox *et al*. [28]	Yes	Yes	Unclear	Unclear	Unclear	Yes	Unclear	Yes	Yes	Unclear
Ilovi *et al*. [33]	Yes	Unclear	Yes	Yes	Yes	Yes	Yes	Yes	Yes	Unclear
Jaffar *et al*. [35]	Yes	Yes	Unclear	Unclear	Yes	Yes	Unclear	Yes	Yes	Unclear
Kagaayi *et al*. [36]	Yes	Unclear	Unclear	Unclear	Yes	Yes	Yes	Yes	Yes	Unclear
Kassa *et al*. [24]	Yes	Yes	Yes	Unclear	Yes	Yes	Unclear	Yes	Yes	Unclear
Martinson *et al*. [37]	Yes	Unclear	Unclear	Unclear	Unclear	Yes	No	Yes	Unclear	High
McGrath *et al*. [29]	Yes	Yes	Yes	Yes	Unclear	Yes	Yes	Unclear	Yes	Unclear
Miiro *et al*. [30]	No	Yes	Yes	Unclear	Yes	Yes	Unclear	Yes	Yes	High
Morpeth *et al*. [31]	Yes	Unclear	Yes	Unclear	Yes	Yes	Unclear	Yes	Yes	Unclear
Tassie *et al*. [32]	Yes	Unclear	Unclear	Unclear	Yes	Yes	Unclear	Yes	Yes	Unclear
Torpey *et al*. [38]	Yes	Yes	Unclear	Yes	Yes	Yes	Yes	Yes	Yes	Unclear

Summary of methodological assessment and risk of bias judgement in included studies and their potential for risk of bias using the QUADAS 2 tool. Each item in the column represents a QUADAS 2 signalling question; each row represents the study and the response as assessed by the authors. A response of: “Yes” means that there was enough data reported in the study paper to provide judgement for low risk of bias. “No” means that the data reported in the study paper had a potential of high risk of bias. “Unclear” means that there was insufficient data reported in the study paper to unable assess the risk of bias. In the overall risk of bias judgement, a study was judged to be at low risk of bias if all signalling questions were answered “Yes.” If a study had one or more signalling questions with a “No” response, it was judged to be at high risk of bias. A judgement of “Unclear” was given where inadequate information to assess risk of bias was available.

**Table 3:** Conduct of WHO clinical staging and CD4 count testing in selected studies

Study	Who performed the WHO clinical staging?	Was WHO Clinical staging assessor pre-trained?	Did assessors use a reference checklist when staging patients?	Did assessors have expert supervision routinely provided?	Were blood samples for CD4 count testing taken after WHO staging? (yes/no)	Were assessors blinded of CD4 count results? (yes/no/unclear)
Baveewo *et al*. [25]	Clinicians^a^	Yes	Yes	NR	Yes	Yes
Boniphace *et al*. [26]	NR	NR	NR	NR	NR	Unclear
Carter *et al*. [27]	Clinicians	Yes	NR	NR	NR	Unclear
French *et al*. [34]	NR	NR	Yes	NR	NR	Yes
Fox *et al*. [28]	NR	NR	NR	NR	NR	Unclear
Ilovi *et al*. [33]	NR	NR	Yes	NR	Yes	Yes
Jaffar *et al*. [35]	Clinician (Doctors)	NR	NR	NR	NR	Unclear
Kagaayi *et al*. [36]	Clinicians	Yes	NR	Yes	NR	Unclear
Kassa *et al*. [24]	Clinician (Doctors)	NR	Yes	NR	NR	Unclear
Martinson *et al*. [37]	Nurses and doctors	NR	NR	NR	NR	Unclear
McGrath *et al*. [29]	Medical assistants^b^	NR	Yes	NR	Yes	Yes
Miiro *et al*. [30]	Clinician (Doctors)	Yes	Yes	NR	NR	Unclear
Morpeth *et al*. [31]	Healthcare workers^c^	Yes	Yes	NR	NR	Unclear
Tassie *et al*. [32]	NR	NR	NR	NR	NR	Unclear
Torpey *et al*. [38]	Clinicians	Yes	NR	NR	Yes	Yes

a Cadre stated as “clinician,” but no further definition given;

b medical assistants – health workers with a medical certificate qualification;

c cadre stated as “health worker,” but no further definition given. NR, not reported.

Three studies [27, 30, 37] were judged to be at an overall high risk of bias. In two of these studies [27, 37], participant flow and timing could have resulted in misclassification bias: the time period between WHO clinical staging and CD4 count measurement was long enough to have potentially affected the participants’ clinical stages, with CD4 counts being measured up to 91 days after staging. In the remaining study [30], patients who were in WHO clinical stage 4 *and* >60 years old were excluded. These patient selection procedures resulted in a judgement of high risk of bias as these exclusion criteria could have compromised index test validity and may mean results are not applicable to the wider population of individuals undergoing WHO clinical staging in routine care.

### Blinding to CD4 count results

Five studies reported that the staging assessors were blinded to the CD4 count results [25, 29, 33, 34, 38] (Table 3). In four of these studies blood for CD4 count measurement was taken after WHO clinical staging [25, 29, 33, 38]. The remaining 10/15 studies did not report when blood for CD4 count was taken, or whether the individuals performing the clinical staging were blinded to the CD4 count results.

### Conduct of WHO clinical staging assessments

#### WHO clinical staging assessors

In eight studies, clinicians performed WHO clinical staging assessment [24, 25, 27, 30, 35–38] (Table 3). Three studies [24, 30, 35] specified that the clinicians were medical doctors, while one study [37] used both nurses and doctors. In six of these eight studies, the clinicians received formal study pre-training in the WHO clinical staging system (Table 3). Two studies reported that other cadres of healthcare workers performed staging [29, 31]: medical assistants in one study [29] and unspecified pre-trained health-care workers in the other [31]. No studies reported the length of training provided.

#### Reference checklist and expert support and supervision

In six studies, staging assessors used reference checklists when assessing patients [24, 25, 29, 30, 33, 34] (Table 3). Only one study [36] reported that WHO clinical staging assessors had regular support and routine supervision from an expert HIV physician during the study period.

### CD4 count methods

Most studies used flow cytometry methods to measure CD4 count for study participants [24–26, 28, 30, 32–38] . One study [31] used the Coulter manual method and in three studies [27, 29, 37], the method was not reported. Only two studies [25, 30] reported that external laboratory quality assurance procedures for CD4 counts were followed. The median CD4 count of participants ranged from 158 to 336 cells/mm^3^.

### Accuracy of the WHO clinical staging

#### CD4 count ≤200 cells/mm^3^

**Figure 2:**
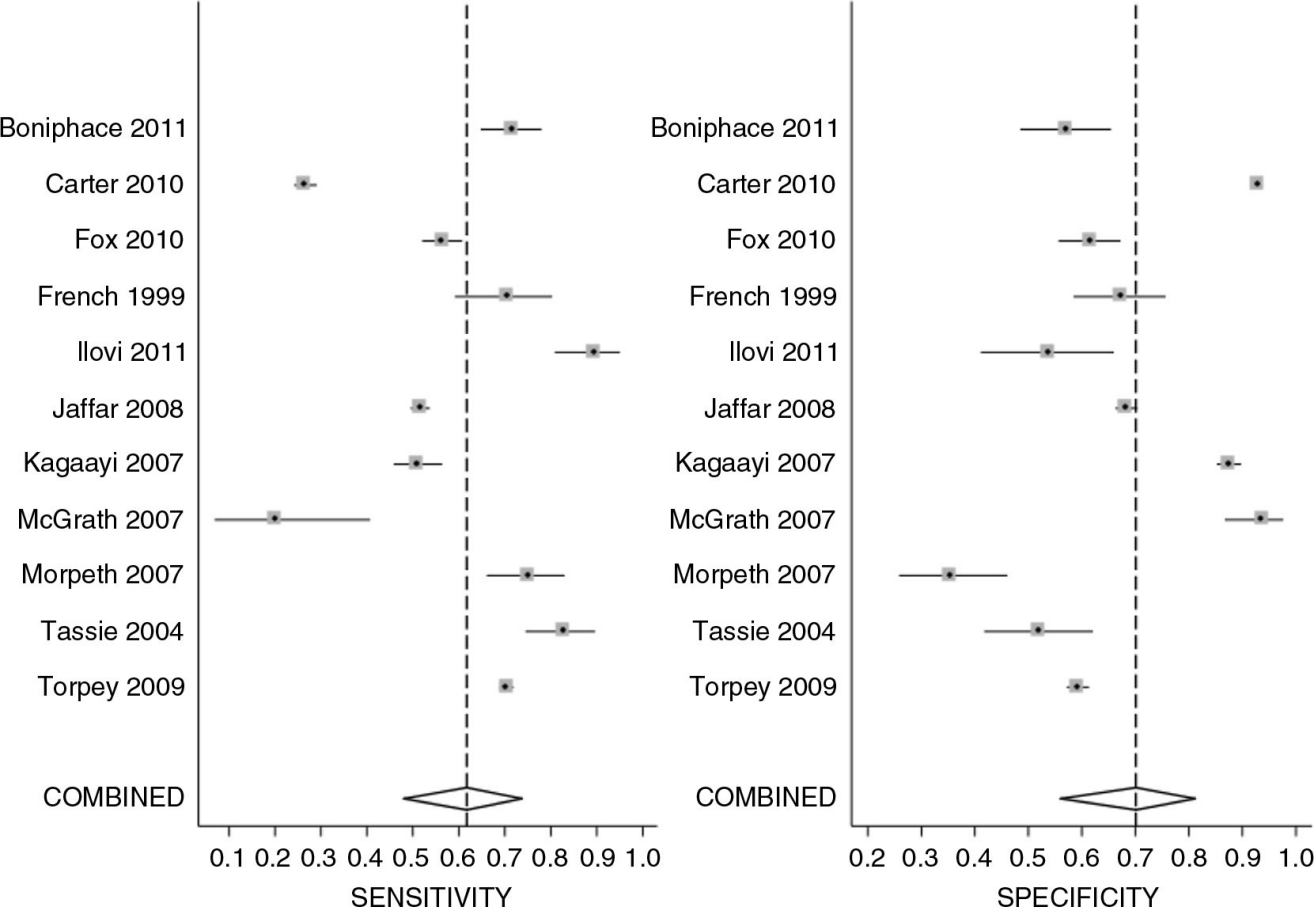
Coupled forest plot of sensitivity and specificity of WHO clinical stage 3 or 4 assessment at CD4 count cut-off value of ≤200 cells/mm^3^. Sensitivity: *Q*=914.26, *p*<0.001; specificity: *Q*=1439.43, *p*<0.001.

In total, 13/15 studies provided data to allow assessment of the diagnostic test performance of WHO clinical staging 3 and 4 at CD4 count threshold of ≤200 cells/mm^3^ (Table 4). At this threshold, sensitivities ranged from 20 to 89%, while specificities ranged from 36 to 93%. Positive predictive values (PPV) ranged from 50 to 74% and negative predictive values (NPV) ranged from 43 to 82% (Table 4). There was strong statistical evidence of heterogeneity between studies for both sensitivity and specificity (sensitivity: *Q*=914.26, *p*<0.001; specificity: *Q*=1439.43, *p*<0.001) (Figure 2).

**Table 4:** Estimates of accuracy of the WHO clinical stage 3 or 4 assessment in identifying CD4 count criteria for ART eligibility

Study	Number true positive	Number false positive	Number true negative	Number false negative	Sensitivity (95% CI)	Specificity (95% CI)	Positive predictive value (95% CI)	Negative predictive value (95% CI)
				**Reference standard: CD4 count ≤200 cells/mm** ^**3**^				
Boniphace *et al*. [26]	137	60	80	54	72% (66–78)	57% (49–66)	70% (63–75)	60% (51–68)
Carter *et al*. [27]	343	337	4408	948	27% (24–29)	93% (92–94)	50% (46–54)	82% (81–83)
Ilovi *et al*. [33]	76	31	36	9	89% (81–95)	54% (41–66)	71% (61–78)	80% (66–89)
French *et al*. [34]	55	40	83	23	71% (59–80)	67% (58–76)	58% (48–67)	78% (69–85)
Fox *et al*. [28]	291	110	176	225	56% (52–61)	62% (56–67)	73% (68–77)	43% (39–49)
Jaffar *et al*. [35]	1163	648	1400	1091	52% (50–54)	68% (66–70)	64% (62–66)	56% (54–58)
Kagaayi *et al*. [36]	186	106	751	178	51% (46–56)	88% (86–90)	64% (58–69)	80% (78–83)
Martinson *et al*. [37] a	–	–	–	–	61%	70%	62%	69%
McGrath *et al*. [29]	5	6	89	20	20% (7–41)	94% (87–98)	45% (21–72)	82% (73–88)
Miiro *et al*. [30] a	–	–	–	–	70%	57%	55%	72%
Morpeth *et al*. [31]	82	60	33	27	75% (66–83)	36% (26–46)	58% (49–65)	55% (42–67)
Tassie *et al*. [32]	88	48	52	18	83% (74–90)	52% (42–62)	65% (56–72)	74% (62–83)
Torpey *et al*. [38]	2548	882	1284	1070	70% (69–72)	59% (56–61)	74% (73–76)	54% (53–57)
Weighted summary					60% (45–73)	73% (60–83)		
				**Reference standard: CD4 count ≤350 cells/mm** ^**3**^				
Baveewo *et al*. [25]	138	15	99	143	49% (43–55)	87% (79–92)	90% (84–94)	40% (84–94)
Carter *et al*. [27]	506	174	3121	2235	18% (17–20)	95% (94–96)	74% (71–78)	58% (57–60)
Ilovi *et al*. [33]	95	14	24	21	82% (73–88)	63% (46–78)	87% (79–92)	53% (39–67)
Jaffar *et al*. [35]	1515	296	851	1640	48% (46–50)	74% (72–77)	84% (82–85)	34% (32–36)
McGrath *et al*. [29]	11	0	69	40	22% (11–35)	100% (95–100)	100% (74–100)	63% (54–72)
Torpey *et al*. [38]	3147	283	691	1663	65% (64–67)	71% (68–74)	92% (90–93)	29% (28–31)
Weighted summary					45% (26–66)	85% (69–93)		
				**Reference standard: CD4 count ≤500cells/mm** ^**3**^				
Carter *et al*. [27]	580	100	1853	3503	14% (13–15)	95% (94–96)	85% (82–88)	34% (33–36)

Table 4 summarizes sensitivity and specificity of each included study at CD4 count cut-off values of either ≤200, ≤350 or ≤500 cells/mm^3^ and the weighted summary estimates of studies at each corresponding CD4 count cut-off.

a Martinson *et al*. [37] & Miiro *et al*. [30] – the studies only presented summary estimates; no raw data to calculate confidence intervals.

**Figure 3:**
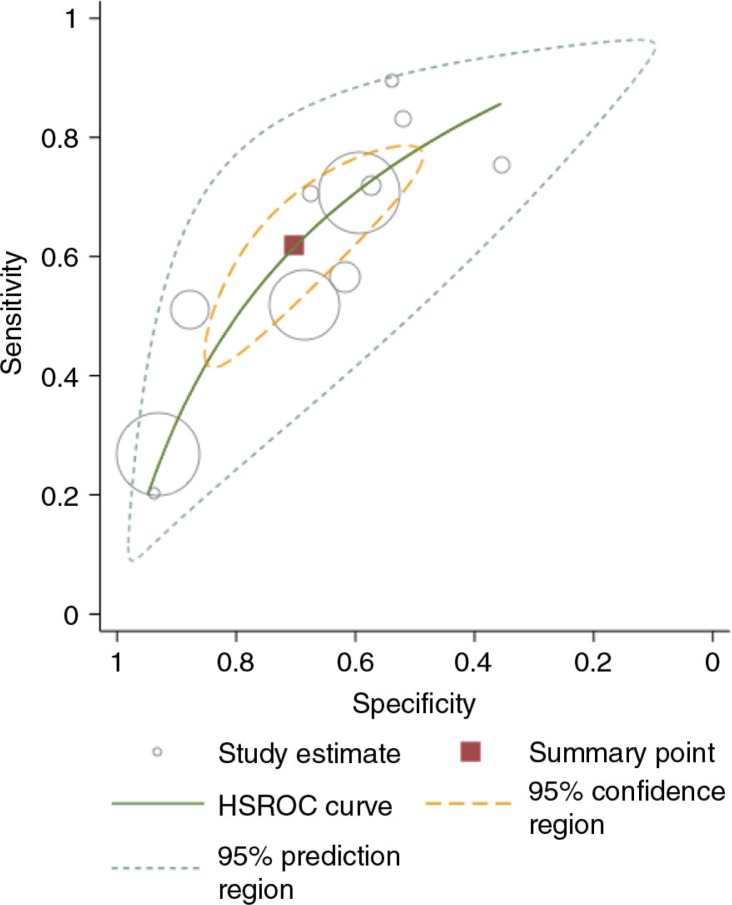
Hierarchical summary receiver operating characteristic (HSROC) of sensitivity verse specificity for the performance of WHO stage 3 or 4 for diagnosing patients with CD4 count ≤200 cells/mm^3^. Each symbol represents a study, with the size of each circle being proportional to the inverse standard error of the sensitivity and specificity respectively. The curves represent the summary receiver operator characteristic curves for WHO clinical stage 3 or 4. The square represents the summary estimate of test performance while the zone outline surrounding it represents the 95% confidence region of the summary estimate.

Eleven out of 13 studies evaluating the accuracy of WHO clinical staging system at CD4 count ≤200 cells/mm^3^ had data available to allow inclusion in the random effect meta-analysis. The pooled sensitivity at CD4 ≤200 cells/mm^3^ was 60% (95% CI: 45–73%) and the pooled specificity was 73% (95% CI: 60–83%), see Figure 3.

Two studies [27, 29] enrolled participants identified through antenatal services (where provider initiated HTC was offered) and a DHS site respectively. In these two studies the sensitivity of WHO clinical staging was low ranging from 20 to 27% at CD4 count cut-off value of 200 cells/mm^3^. In contrast, the sensitivity of the staging was relatively high ranging from 80 to 83% in the two studies that enrolled hospital inpatients [33, 38].

#### CD4 count ≤350 cells/mm^3^

**Figure 4:**
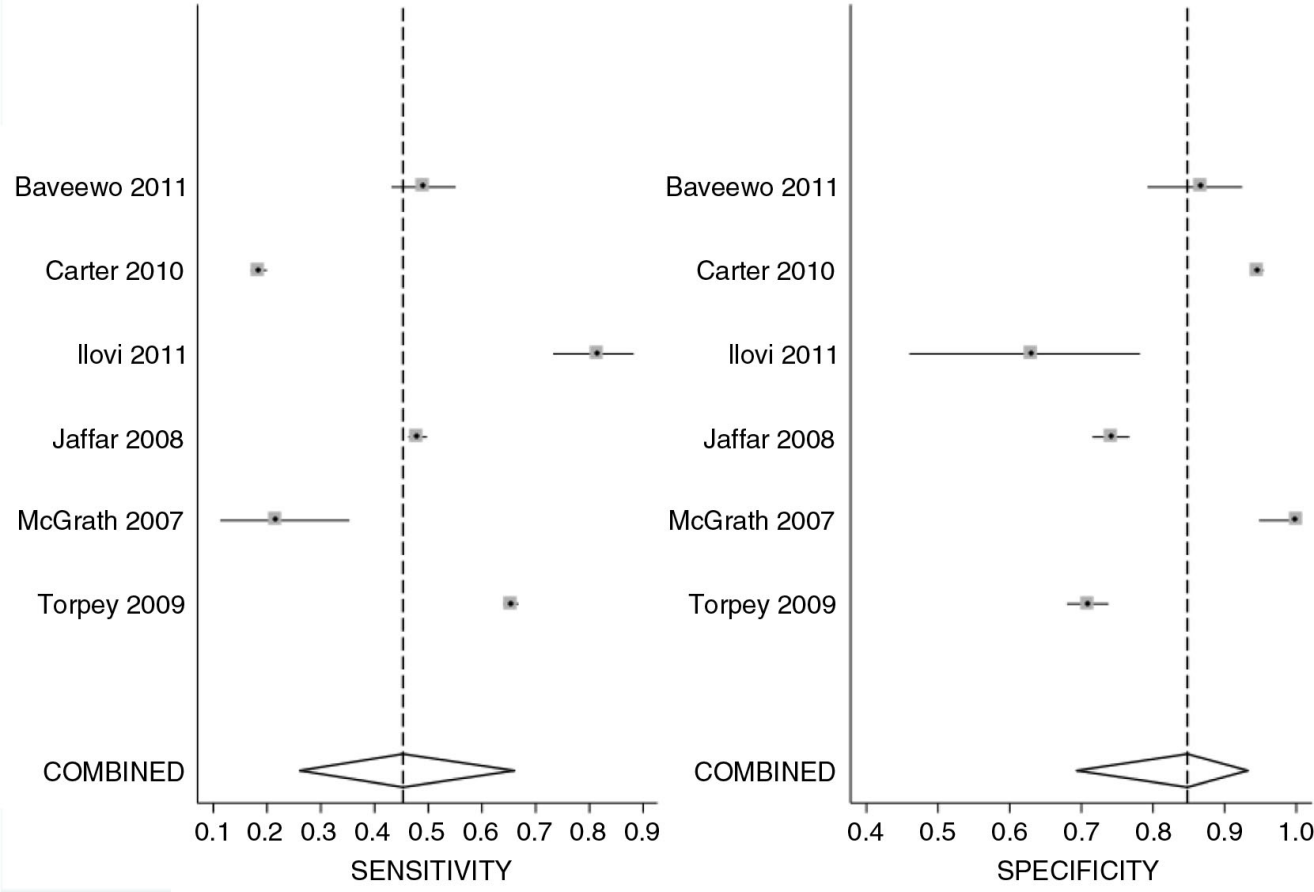
Coupled forest plot of sensitivity and specificity of WHO clinical stage 3 or 4 assessment at CD4 count cut-off value of <350 cells/mm^3^. Sensitivity *Q*=1607.31, *p*<0.001; specificity *Q*=896.70, *p*<0.001.

**Figure 5:**
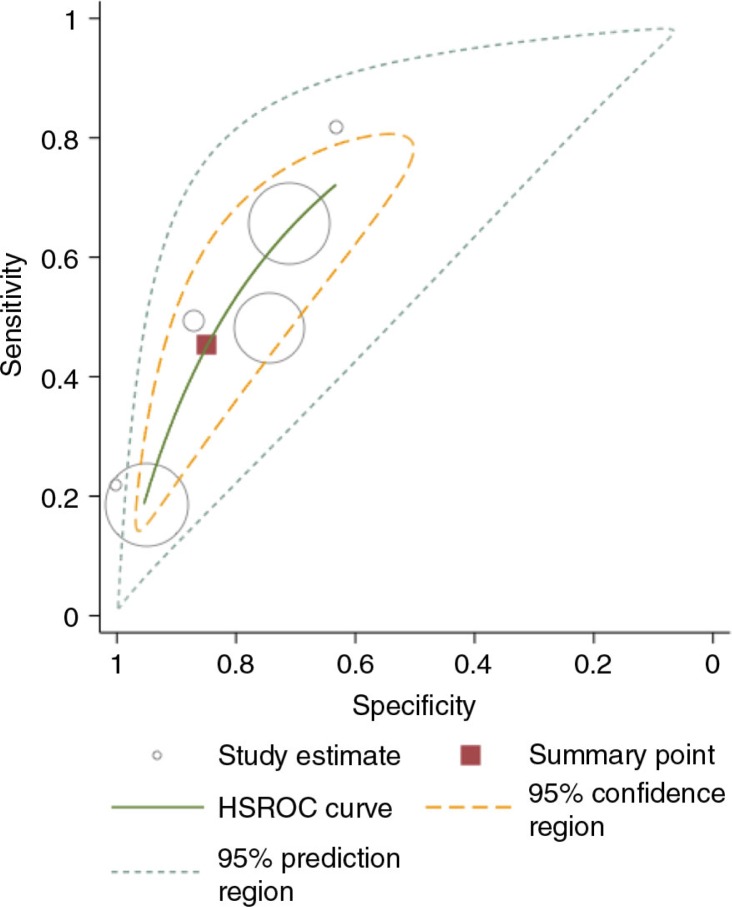
Hierarchical summary receiver operating characteristic (HSROC) of sensitivity verse specificity for the performance of WHO stage 3 or 4 for diagnosing patients with CD4 count ≤350 cells/mm^3^. Each symbol represents a study, with the size of each circle being proportional to the inverse standard error of the sensitivity and specificity respectively. The curves represent the summary receiver operator characteristic curves for WHO clinical stage 3 or 4. The square represents the summary estimate of test performance while the zone outline surrounding it represents the 95% confidence region of the summary estimate.

Six studies used CD4 count ≤350 cells/mm^3^ as a threshold for ART eligibility [25, 27, 29, 33, 35, 38] (Table 4). The sensitivity of the WHO clinical staging ranged from 18 to 82%, and specificity ranged from 63 to 100%. The PPV ranged from 74 to 100% while the NPV ranged from 29 to 63%. There was heterogeneity across studies for both sensitivity and specificity, as shown in Figure 4 (sensitivity *Q*=1607.31, *p*<0.001; specificity *Q*=896.70, *p*<0.001). Meta-analysis of these six studies [25, 27, 29, 33, 35, 38], showed a pooled sensitivity of 45% (95% CI: 26–66%, *p*<0.001) and specificity of 85% (95% CI: 69–93%, *p*<0.001) (Figure 5).

#### CD4 count ≤500 cells/mm^3^

One study [27] evaluated the diagnostic accuracy of WHO clinical stage 3 and 4 at a CD4 count threshold of ≤500 cells/mm^3^, showing a sensitivity of 14% (95% CI: 13–15%) and specificity of 95% (95% CI: 94–96%), see Table 4.

## Discussion

The main finding of this systematic review and meta-analysis assessing accuracy of WHO stage 3 or 4 assessment compared to CD4 count strata was the low sensitivity of the WHO clinical staging system, with poor ability to distinguish between ART eligible and ineligible individuals. The weighted summary estimates show that sensitivity worsened as CD4 count threshold increased. This may lead to patients not starting treatment despite being eligible for ART. If CD4 counts are not available, the clinical and public health benefits of moving to a higher threshold will be lost [4, 39, 40].

In settings where laboratory-based CD4 measurements are unavailable, alternatives are therefore required that provide a more accurate ART eligibility assessment [41–43]. The roll out of point-of-care (POC) CD4 tests has gone some way to meet this need; more than 2500 machines were in use in 41 low- and middle-income countries at the end of 2012 [1]. Notwithstanding this rapid scale-up, currently available POC CD4 count machines are costly and difficult to maintain in many low-income settings [5]. Using low-cost CD4 count testing to guide ART eligibility has been proven to be very cost-effective compared to WHO clinical staging at both CD4 count threshold of ≤200 and ≤350 cells/mm^3^. Low cost CD4 testing compared to WHO clinical staging improves both the quantity and quality of life and has an incremental cost-effectiveness ratio (ICER) of $80 per life year gained and $85 per QALY gained [44].

Task shifting of ART care to non-physician clinicians has been a critical strategy to support scale-up in settings where human resources are scarce, and is recommended by the latest WHO guidelines [1]. In two studies [30, 35] included in this review, physicians performed clinical staging assessments while medical assistants performed clinical staging in one study [29]. However, it is difficult to assess the effect of task shifting on the performance of WHO clinical staging as studies were done in different settings. A study done in Mozambique to assess the performance of non-physician clinician *Tecnicos de Medicina* in their ability to perform clinical staging in the outpatient clinics found that the non-physician clinicians were only able to assign a correct WHO clinic stage in 37.5% of the patients [45]. These findings highlight the complexities of the WHO clinical staging system, which has case-definitions that require in-depth medical knowledge, experience and ability to interpret laboratory diagnostic results. As such, while the need for rapid clinical staging continues, alternatives simplified approaches need to be developed.

Despite its poor performance described here, retaining WHO clinical staging as an ART eligibility assessment tool has a number of benefits if properly performed, including in settings where CD4 cell count is available. WHO clinical staging provides health workers an opportunity to perform a thorough assessment and physical examination and to screen patients for tuberculosis and other conditions, including those that may lead to an immune reconstitution syndrome (IRIS) during ART initiation [46]. Removal of WHO clinical staging completely may risk missed opportunities for identification and treatment (or provision of chemoprophylaxis) for opportunistic infections. In addition, in settings with limited availability of CD4 counts, like in Malawi where only 11% of ART centres have a functional CD4 count machine, WHO staging provides a measure of programme performance since it identifies patients who have presented late to the clinical services [6].

There were a number of strengths and limitations to this study. Strengths include a broad search strategy and the use of rigorous systematic review methodology guided by a registered protocol. A limitation of this review is the risk of publication bias resulting from the exclusion of non-English language studies, restricted geographical range and the limited number of databases searched.

There are also limitations relating to the quality of available evidence. Notably, only one of the 15 included studies was judged to be at overall low risk of bias [25], with a further three at high risk of bias [27, 30, 37]. Potential misclassification bias was noted in two studies, with delays between performing the index and reference test, and one study restricted participant eligibility for inclusion so that patients with AIDS-defining conditions were excluded, limiting applicability. Moreover, the majority of studies did not fully describe conduct of WHO clinical staging procedures, raising concerns that there may have been potential for information and systemic biases that could mean the accuracy of the WHO clinical staging system was over-, or under-estimated. Conduct of the reference test (CD4 count) may have been suboptimal as only two studies [25, 30] reported following external quality assurance procedures.

Study estimates for sensitivity and specificity were highly heterogeneous. However, with the small numbers of studies we were unable to perform sensitivity analysis to examine the effect of: year of study; individual that performed WHO clinical staging; site of evaluation; risk of bias; or blinding to CD4 count result on the accuracy of WHO clinical staging. Although random-effects models were used to account for heterogeneity, pooled estimates should be interpreted with caution. Attempts were made to assess the performance of WHO staging system between studies that enrolled ART-naïve participants and those that enrolled participants reinitiating treatment. However, no studies provided such data.

Only 15 studies were identified over a period of 15 years. This is surprising considering how widely the WHO clinical staging system is used in sub-Saharan Africa. Although a systematic literature search was undertaken, identifying all studies that may have reported on accuracy of the WHO clinical staging system was challenging as it was often not the main focus of the study. As such, it is possible that eligible studies have been inadvertently excluded.

Since the first WHO guidelines for ART initiation in resource-limited settings were published in 2002, the lack of diagnostic capacity has been recognized and all subsequent guidelines have stated that lack of laboratory testing should not act as a barrier to ART initiation. This principle remains a critical cornerstone of the public health approach. However, continued efforts are needed to improve access to key diagnostic tests such as CD4 cell count and viral load measurement to accurately ascertain the need for ART, particularly as WHO recommendations for ART change to include patients in earlier stages of HIV infection.

This study has shown that the performance of the WHO clinical staging system is insufficiently accurate compared to the current gold standard ART eligibility assessment of CD4 count, with the consequence that considerable numbers of patients will be unnecessarily delayed or missed in initiating ART if clinical stage alone is used. The performance of WHO clinical staging varies across different study population with hospital-based studies showing a relatively higher sensitivity than community-based studies.

These findings strongly support calls for access to cheap, quality assured CD4 count testing methods for as long as ART initiation decisions are based on criteria other than seropositivity. An alternative approach to overcoming this problem that is increasingly being considered is to remove the need for clinical or immunological eligibility and initiate ART in all people living with HIV irrespective of disease status.
